# Impact of Excessive Postural Tachycardia on Disability in Youth with Orthostatic Intolerance

**DOI:** 10.1016/j.jpeds.2026.115042

**Published:** 2026-02-17

**Authors:** Kirti Sivakoti, Olivia Parnell, Corinne Espinoza, Casey Tak, Philip Fischer, Julie Shakib, Stanley Brewer, Deirdre Caplin, Shauna Skog, Melissa Cortez

**Affiliations:** Division of General Pediatrics, University of Utah and Primary Children’s Hospital, Salt Lake City, UT, 1-Mayo Clinic, Rochester, MN

## Abstract

**Objective:**

To compare Functional Disability Inventory (FDI) between adolescents with chronic orthostatic intolerance (COI) and postural orthostatic tachycardia syndrome (POTS).

**Study design:**

This cross-sectional study included adolescents ≤19 years with orthostatic symptoms >3 months referred to a tertiary pediatric autonomic clinic (August 2024–March 2025) and classified as POTS or COI based upon active stand testing. Self- and parent-reported FDI scores were collected. Group comparisons used chi-square and Wilcoxon–Mann–Whitney tests. Univariable and multivariable linear regression models compared FDI scores, adjusting for age, sex, and body mass index. Adjusted means were estimated using post-fit marginal estimation.

**Results:**

Ninety-two adolescents were included (46 POTS, 46 COI). Groups were similar in age, sex, and body mass index, but differed in heart rate response by definition. Self-reported FDI scores did not differ between COI and POTS in unadjusted (22.9 vs 22.4; *P* = .81) or adjusted analyses (adjusted mean difference 1.0, 95% CI −3.0 to 4.9; *P* = .63). Parent-reported FDI scores were also comparable in unadjusted (23.1 vs 22.3; *P* = .70) and adjusted analyses (adjusted mean difference 0.4, 95% CI −3.4 to 4.2; *P* = .85). Differences were well below the prespecified threshold for clinical significance.

**Conclusion:**

There was no evidence for a difference in functional disability in adolescents with COI, regardless of tachycardia-based POTS classification. This suggests that heart rate response is not associated with functional differences in pediatric orthostatic intolerance.

Chronic orthostatic intolerance (COI) refers to a group of conditions in which upright posture triggers a wide range of symptoms, including dizziness, fatigue, cognitive fog, and palpitations, among others, persisting for at least 3 months.^1,2^ Postural orthostatic tachycardia syndrome (POTS) represents a specific subset of COI, distinguished by an exaggerated heart rate (HR) response, defined in adolescents as an increase of ≥40 beats per minute (bpm) or an upright HR exceeding 120 bpm (without accompanying orthostatic hypotension), while those with HR increase <40 bpm remain classified as COI.^1,3–8^ These diagnostic criteria were established based on adolescent tilt table testing data and formalized in a 2015 Heart Rhythm Society consensus statement.^1^

Although awareness of POTS has grown, COI patients who do not meet HR criteria remain isolated and face ongoing uncertainty and limited access to care. Their experiences have rarely been captured in literature, and little is known about their functional impairment, treatment options, or clinical outcomes. In our practice, we see these patients frequently encounter diagnostic ambiguity, are excluded from evidence-based interventions, and face repeated dismissal, which can worsen functional decline.^9^ Even when their symptoms and disability match those of POTS pers, many lack a formal diagnosis, individualized treatment plan, or access to interventions such as volume expansion or cardiac reconditioning. This combination of exclusion and isolation delays functional improvement and leaves patients without he support necessary to manage their condition. These challenges demonstrate the urgent need to recognize and address the needs of COI patients who fall outside conventional POTS criteria defined through HR.

At our interdisciplinary pediatric dysautonomia center, we observed that adolescents with COI and subthreshold tachycardia are more common than those meeting full POTS criteria, and based on clinical observation, appear to experience functional difficulties similar to their POTS peers. We hypothesized that functional impairment would be comparable between patients with and without excessive tachycardia, regardless of HR response to standing. To test this, we compared perceived functional disability using the Functional Disability Inventory (FDI), a validated, patient-centered tool capturing difficulty in school, sports, and social participation. Assessing functional disability from both patient and parent perspectives provides a holistic view, which is particularly important in community and resource-limited settings where access to advanced autonomic testing is often delayed or unavailable.

## Methods

This cross-sectional study was conducted at the Pediatric Dysautonomia Program at Primary Children’s Hospital between August 2024 and March 2025 to evaluate functioning in patients diagnosed with COI and POTS. The study is reported in accordance with the STROBE (Strengthening the Reporting of Observational Studies in Epidemiology) guidelines.

The study protocol was reviewed and approved by the Institutional Review Boards of Intermountain Healthcare and the University of Utah (IRB # 00 169 137), both of which granted a waiver of informed consent due to the study’s retrospective nature. Parental or guardian consent and child assent were not required under the waiver, as only deidentified data from existing medical records were analyzed. All procedures complied with institutional and national ethical guidelines for research involving human participants.

Race and ethnicity were self-reported or reported by parents/guardians at clinic registration, with race categorized as American Indian or Alaska Native, Asian, Black or African American, Native Hawaiian or Other Pacific Islander, or White and ethnicity categorized as Hispanic or non-Hispanic.

Data were obtained from the Research Electronic Data Capture clinical database, which houses demographic and symptom-related information from the electronic health records of patients evaluated within the Dysautonomia Program.^10^ Research Electronic Data Capture is a secure, web-based platform providing (1) an intuitive interface for validated data capture; (2) audit trails for tracking data manipulation and export; (3) automated export procedures for seamless downloads to common statistical packages; and (4) integration and interoperability with external sources.^10,11^

Patients were eligible if they were 19 years or younger at the time of evaluation in our Dysautonomia Program and had symptoms of COI lasting longer than 3 months. Patients were included consecutively through chart review to ensure a representative sample. All 92 patients included identified as White and non-Hispanic. Although COI is more frequent than POTS in our program, equal-sized groups were included for balanced comparison, with the study period for POTS patients extended to achieve statistical power.

At initial intake, all patients underwent a comprehensive evaluation by pediatric autonomic specialists (K.S., S.B., S.S.) including detailed medical history, physical examination, and functional assessment. The outcome, functional disability was assessed using the parent- and self-reported FDI, a validated 15-item questionnaire measuring perceived impairment across mobility, self-care, physical comfort, social participation, and psychological functioning. Responses are scored on a 5-point Likert scale (0 = no trouble to 4 = impossible) and summed to a total score (0–60), with higher scores indicating greater disability.^12,13^ Both parent and child forms were completed for all participants.

Autonomic function was assessed using a standardized 10-minute active stand test. Baseline HR, blood pressure (BP), and oxygen saturation were recorded supine. Patients then stood motionless for 10 minutes, avoiding leaning or external support, with HR, BP, and oxygen saturation measured once per minute. The highest standing HR was used to calculate ΔHR (difference from supine), and symptoms during the test were documented. Patients with ΔHR ≥40 bpm were classified as POTS, while those with ΔHR <40 bpm but experiencing orthostatic symptoms were classified as COI. Patients with HR near the threshold (eg, >35 bpm) were included in the COI group even if an ICD-10 POTS diagnosis was present. All 10-minute active stand tests were documented in scanned reports within the electronic health record, allowing verification of autonomic measurements and cross-checking of ICD-10 diagnostic codes. Orthostatic hypotension was defined as a decrease in systolic BP > 20 mmHg or diastolic BP > 10 mmHg; no patients met these criteria, so no exclusions were made.

Patients taking medications known to affect autonomic function, including volume-expanding agents (fludrocortisone), vasoconstrictors (midodrine), beta-blockers, selective serotonin reuptake inhibitors, tricyclic antidepressants, and stimulants, were not excluded. Medication use at the time of evaluation was minimal and was not specifically analyzed, although its potential impact on autonomic function remains an area for future research.

### Statistical Analysis

Statistical analyses were performed using Stata version 18. We compared patient characteristics between COI and POTS on potential confounding variables using chi-square test for categorical variables and Wilcoxon-Mann-Whitney test for continuous variables. We assessed normality of continuous variables using the Shapiro-Wilks test, and found no violations of normality in our outcome variables; however, both continuous patient characteristics had slight departures from normality, which is why we used the Wilcoxon-Mann-Whitney test for those comparisons. We fit both univariable and multivariable linear regression models on the primary outcomes of self-reported and the parent-reported FDI questionnaires. The adjusted means from the multivariable model were estimated using post-fit marginal estimation.

The study was adequately powered to detect a clinically meaningful difference between POTS and COI groups. Based on prior research, a 7-point difference in FDI scores was considered clinically important.^14^ Assuming a moderate effect size, with alpha = 0.05 and power = 0.80, a minimum of 34 patients per group was required. Our sample included 46 patients per group (92 total).

## Results

The study cohort included 92 adolescents evaluated in our Dysautonomia Program, all of whom identified as White and non-Hispanic, reflecting the demographics of our clinical population. No exclusions were made based on race or ethnicity. The cohort was evenly divided between patients meeting POTS criteria (n = 46) and those with COI without excessive tachycardia (n = 46).

Table I presents the demographic and physiological characteristics of the total cohort (n = 92) and the 2 subgroups of adolescents with COI: those meeting POTS criteria (n = 46) and those without excessive tachycardia (COI, n = 46). The groups were comparable in terms of age (16.2 ± 1.9 vs 16.2 ± 1.5 years, *P* = .81), sex distribution (74% vs 83% female, *P* = .31), and body mass index (BMI) (22.0 ± 4.2 vs 24.5 ± 7.8 kg/m^2^, *P* = .19). As expected, the groups differed in HR response by definition, with the POTS group demonstrating a higher mean maximum HR during active stand (118.5 ± 12.5 vs 104.9 ± 12.9 bpm) and a greater increase from supine to standing (46.3 ± 6.2 vs 28.4 ± 8.2 bpm). See Table I below.

For the outcome of self-reported FDI, the unadjusted mean for COI patients was 22.9 (95% CI: 20.0-26.0) and for POTS patients was 22.4 (95% CI: 19.5-25.4), and the unadjusted mean difference was −0.5 (95% CI: −4.7, 3.7) (*P* = .81) (Table II). For the outcome of self-reported FDI, the adjusted mean for COI patients was 22.2 (95% CI: 19.4-25.0) and for POTS patients was 23.2 (95% CI: 20.4-25.9), and the adjusted mean difference was 1.0 (95% CI: 3.0, 4.9) (*P* = .63), after adjusting for sex, age, and BMI (Table II).

For the outcome of parent-reported FDI, the unadjusted mean for COI patients was 23.1 (95% CI: 20.3-25.9) and for POTS patients was 22.3 (95% CI: 19.5-25.1), and the unadjusted mean difference was −0.8 (95% CI: −4.7, 3.2) (*P* = .70) (Table II). For the outcome of parent-reported FDI, the adjusted mean for COI patients was 22.5 (95% CI: 19.8-25.2) and for POTS patients was 22.9 (95% CI: 20.2-25.5), and the adjusted mean difference was 0.4 (95% CI: −3.4, 4.2) (*P* = .85), after adjusting for sex, age, and BMI (Table II).

These findings demonstrate comparable functional impairment between groups despite differences in HR response.

## Discussion

In both self-reported and parent-reported FDI, there were no statistically significant differences between patients with POTS and those with COI. Although we did not prespecify that we were going to perform equivalence testing, the 95% CIs around the mean difference were well within ± 7 points on the FDI questionnaire.^15,16^ In previously published literature, a difference of 7 points on the FDI is considered a clinically meaningful difference. So even though the POTS and COI, by definition, do not overlap by the HR criteria, we did not find evidence for a difference in functional impairment.

The clinical implications of these findings are substantial. Patients in both the POTS and COI groups endorsed a high symptom burden and significant disruption in daily life. Relying solely on HR thresholds to define illness risks excluding a cohort of patients who are functionally impaired but do not meet the ≥40 bpm increase used to diagnose POTS. These patients are often told that “nothing is wrong” in community settings, simply because they don’t meet the criteria, and as a result, their access to care is delayed or denied. Meanwhile, their symptoms persist, their functional capacity declines, and their academic and social participation suffers.^9^

Regardless of diagnostic label, these patients present with similar degrees of functional impairment. In terms of care, patients with COI who do not meet formal POTS criteria (so HR on active stand test is < 40 bpm) warrant the same symptom-based, supportive strategies, despite the absence of standardized diagnostic pathways. Treatment should therefore be guided by functional impairment rather than HR thresholds and center on early education, hydration and salt optimization, compression, physical reconditioning, and targeted supportive therapies to prevent ongoing decline and delays in care.^17^ A function-based approach facilitates earlier access to supportive resources such as physical therapy, behavioral health, academic accommodation, and education on hydration, compression, and exercise reconditioning. This model gives providers a framework to move forward confidently in the absence of a specific diagnosis and prevents unnecessary repeat visits from patients seeking further evaluation that is unlikely to change management.

Recent adult literature on long-COVID describes a POTS-like autonomic syndrome marked by COI, tachycardia, fatigue, pain, and cognitive symptoms, with some patients demonstrating delayed worsening months after the acute infection. Although these data are largely derived from adult cohorts, we are increasingly seeing similar postinfectious autonomic symptom patterns in pediatric patients. The substantial overlap between long-COVID–related autonomic symptoms and POTS/COI further supports a function-based, symptom-driven approach to care rather than strict reliance on HR thresholds alone. Early recognition of functional impairment and timely initiation of supportive, rehabilitative, and when appropriate pharmacologic interventions may represent a useful management paradigm for patients with COI, including those with post-COVID autonomic symptoms.^18,19^

There are few scenarios in which HR response alone should dictate treatment (eg, the use of medications such as beta-blockers, which may be more appropriate in cases of excessive tachycardia).^20,21^ However, for the majority of interventions particularly those involving behavioral and rehabilitative strategies, a unified treatment framework can and should be applied to both POTS and COI populations.^22–24^

We chose the active stand test for this study because it is accessible, feasible, and available in outpatient and primary care settings. This enhances the real-world applicability of our findings. Tilt table testing may offer greater diagnostic precision, especially in distinguishing POTS from controls, as demonstrated in recent work by Stewart and Medow.^25^ Their data suggest that the current diagnostic cut-offs may be inappropriate for active stand testing and may explain why standing appears less specific. Rather than abandoning active stand as a diagnostic tool, clinical utility could be improved by adjusting HR thresholds based on the method used.

This study has some limitations. We did not assess longitudinal outcomes or treatment response, so we cannot comment on whether initial functional disability predicts long-term recovery or persistence of symptoms.

Another limitation is that we did not review or include HR-controlling medication use as a co-factor in the regression analysis. This factor may influence HR measurements and could be considered in future analyses.

Other limitations of this study include the lack of a matched control group. This observational design was dictated by the nature of the available data.

A fourth limitation is that, although POTS in adolescents is defined as an increase of ≥40 bpm or an upright HR exceeding 120 bpm without orthostatic hypotension, this definition does not account for diurnal variability in autonomic function.^26^ HR and autonomic activity can fluctuate throughout the day, potentially affecting the magnitude of orthostatic HR changes.^27^ In our study, active stand testing was performed during routine clinic hours without standardizing for time of day. Although this may introduce some variability in measured HR responses, it reflects real-world clinical practice, where patients are seen at different times. Including patients across typical clinic hours enhances the representativeness of our sample and improves generalizability, capturing functional impairment and autonomic responses as they occur in everyday outpatient settings rather than under controlled laboratory conditions.

Future studies should aim to validate these findings in larger, multicenter cohorts and incorporate longitudinal follow-up to examine the trajectory of functional impairment over time. Incorporating patient-reported outcome measures and qualitative data could provide further insight into the lived experience of children and adolescents with POTS and COI. In addition, studies with objective physiologic measures and similar study with a matched control group can be conducted.

## Figures and Tables

**Table I.** Patient characteristics

Characteristic	Total sample [n = 92]	POTS [n = 46]	COI [n = 46]	*P* value
Gender, n (%)				.31
Female	72 (78)	34 (74)	38 (83)	
Male	20 (22)	12 (26)	8 (17)	
Age, yr				.81
Mean ± SD	16.2 ± 1.7	16.2 ± 1.9	16.2 ± 1.5	
Median (IQR)	16.5 (15.5-17.4)	16.5 (15.5- 17.5)	16.6 (15.5-17.1)	
BMI, kg/m^2^				.19
Mean ± SD	23.3 ± 6.3	22.0 ± 4.2	24.5 ± 7.8	
Median (IQR)	22.0 (19.2-24.8)	21.1 (19.0-24.5)	22.7 (19.7-26.0)	N/A*
Active stand HR, mean SD, bpm				
Top	111.7 ± 14.4	118.5 ± 12.5	104.9 ± 12.9	
Bottom	74.4 ± 12.9	72.2 ± 11.7	76.5 ± 13.8	
Difference	37.4 ± 11.5	46.3 ± 6.2	28.4 ± 8.2	

* N/A = POTS and COI groups are different by definition.

**Table II.** Univariable and multivariable linear regression models (n = 92)

	Univariable model			Multivariable model		
Predictor	Unadjusted B	95% CI	*P* value	Adjusted B	95% CI	*P* value
Outcome: Self-reported FDI						
Type of autonomic disease						.63
COI	Ref			Ref		
POTS	−0.5	(−4.7, 3.7)	.81	1.0	(3.0, 4.9)	
Outcome: Parent-reported FDI						
Type of autonomic disease						.85
COI	Ref			Ref		
POTS	−0.8	(−4.7, 3.2)	.70	0.4	(−3.4, 4.2)	

Adjusted B = Regression coefficient (mean difference) after adjusting for gender, age, and BMI.

## Abbreviations

BMI: Body mass index
BP: Blood pressure
BPM: Beats per minute
COI: Chronic orthostatic intolerance
FDI: Functional Disability Inventory
HR: Heart rate
POTS: Postural orthostatic tachycardia syndrome
